# Dramatic Course of Paediatric Cryptogenic Febrile Infection-Related Epilepsy Syndrome with Unusual Chronic Phase Presentation—A Case Report with Literature Study

**DOI:** 10.3390/brainsci11081030

**Published:** 2021-08-02

**Authors:** Natalia Rachfalska, Jerzy Pietruszewski, Justyna Paprocka

**Affiliations:** 1 Students’ Scientific Society, Department of Pediatric Neurology, Faculty of Medical Sciences in Katowice, Medical University of Silesia, 40-752 Katowice, Poland; n.rachfalska@gmail.com; 2 Department of Pediatric Neurology, Faculty of Medical Sciences in Katowice, Medical University of Silesia, 40-752 Katowice, Poland; jpietruszewski@sum.edu.pl

**Keywords:** FIRES, NORSE, RSE, refractory status epilepticus

## Abstract

Febrile Infection-Related Epilepsy Syndrome (FIRES) is a catastrophic, extremely rare epileptic encephalopathy. It strikes previously healthy school-aged children and is usually cryptogenic. Its dramatic onset with refractory status epilepticus is always preceded by a nonspecific febrile illness. The seizure activity in FIRES may last for several weeks with little to no response to antiepileptic treatment, usually resulting in the usage of anaesthetics. This acute phase is followed by a chronic, refractory epilepsy and cognitive deficit, that persist for the rest of the patient’s life. Still to this day no definite cause has been described. In this study we review the current finding in FIRES and describe a case of a 4-year-old patient with a dramatic course of the acute phase in FIRES and unusual presentation of the chronic phase, which is dominated by extrapyramidal symptoms such as dystonia. This case highlights that the clinical presentation of FIRES may differ from those frequently described in literature.

## 1. Introduction

Febrile Infection-Related Epilepsy Syndrome (FIRES) is a catastrophic epileptic encephalopathy affecting school-aged children. Its occurrence is extremely rare, but often fatal nonetheless. It is all the more devastating given that FIRES develops in previously healthy individuals. The aetiology of this phenomenon is still to be discovered, making it a matter of utmost priority for researchers around the world.

FIRES-like syndrome was first described by Lyon et al. in 1961 as “acute encephalopathy of obscure origin in infants and children” [1]. The clinical presentation matching with FIRES has been later described with many names, such as DESC (devastating epileptic encephalopathy in school-aged children) [2], AERRPS (acute encephalitis with refractory, repetitive partial seizures) [3], idiopathic catastrophic epileptic encephalopathy presenting with acute onset intractable status [4]. The name as we know now has been suggested by Van Baalen et al., in 2010 [5]. Nowadays FIRES is considered to be a sub-category of New Onset Refractory Status Epilepticus (NORSE), which is described as a clinical presentation of refractory status epilepticus that occurs in patients without active epilepsy nor with any signs of structural, toxic or metabolic damage to the brain, that can be ruled out in the first 72 h. NORSE, however, includes patients with viral and autoimmune diseases, even if no specific autoantibody has been identified. It is noteworthy that NORSE is not a diagnosis per se, but rather a clinical manifestation of other neurological condition [6]. The difference in FIRES and NORSE is the presence of fever, which is usually attributed to FIRES. Until recently FIRES was thought to be a paediatric form of NORSE, but these days it is considered that both children and adults may develop either FIRES or NORSE [6,7,8].

In this article the authors will focus mostly on FIRES in children, as it is fundamental for the understating of this case study.

We describe a 4-year-old patient with a catastrophic and abrupt course of cryptogenic FIRES with atypical chronic phase presentation, dominated by dystonia. The patient did not respond to any implemented treatment and developed severe encephalopathy.

## 2. Clinical Presentation of FIRES

FIRES typically develops in children between the ages of 3 and 15 years old with median age at onset of around 6.5 years [5]. The majority of paediatric patients are male [3,5,9,10]. It is extremely rare, with its estimated incidence being 1 in 1,000,000 [11]. Children affected by FIRES are usually previously healthy with normal psychomotor development. The family history is usually uninformative and no ethnic predisposition has been observed [2,12].

In FIRES the onset of refractory status epilepticus is subsequent to a febrile infection with fever starting between 24 h and 2 weeks prior. However, fever is not necessarily present at the time the seizures begin—it is important to notice that FIRES is not simply febrile status epilepticus as in prolonged febrile convulsions. The fever itself was mostly associated with unremarkable upper respiratory tract infection and gastrointestinal problems to a lesser extent [2,5,9]. This stage is called the infectious phase and is thought to be the period in which a subclinical epileptogenesis occurs [12].

The latter acute phase begins with the onset of the seizures. At first they are brief with their frequency increasing gradually in a few hours up to a day, evolving into refractory status epilepticus (RSE) with even 100 seizures a day [13,14]. RSE is defined as status epilepticus persisting despite administration of at least two appropriately selected and dosed parenteral medications including benzodiazepine [6]. Treatment with anti-epileptic drugs (ASMs) is usually disappointing with patients receiving up to 15 different medications without success [2,5,9]. This acute phase lasts from 1 to 12 weeks with a median length of 3 weeks and results in death in up to 25% of cases [5,15]. It generally requires a long hospitalisation in the intensive care unit (ICU).

With the decrease of seizure frequency, the status epilepticus finally ceases, and thus the chronic phase occurs. The long-term outcome is overall poor [2,9]. Most of the patients that survive the acute phase are left with refractory epilepsy that occurs right after or not further than 3 months from the end of the acute phase [3,9,16,17] which is distinguishing from epilepsy due to encephalitis or trauma [12,18]. The seizures are mostly of the same type as those seen in status epilepticus period and involve the same regions [2,3,9,14]. They often occur in clusters every 2–4 weeks with median frequency around 3 per month [2,14]. Moreover, most of the patients are left with cognitive disfunctions to some extent. In the majority of the cases those affected temporal and frontal lobe, including memory deterioration, speech impairment, functional disability and emotional instability. However, any remediation in these children is often reversed after another cluster of seizures occurs [14]. Some of the patients ended up in a vegetative state [3,4,5,14].

## 3. Aetiology of FIRES

As highlighted before, FIRES and NORSE are manifestations of other conditions, most of which are unknown due to no specific findings in clinical studies [13]. This so-called cryptogenic NORSE makes up 50% of the cases in adults. The most frequent conditions notorious for causing status epilepticus (SE) are auto-immune and inflammatory encephalitis (40%), infectious encephalitis (10%) and genetic disorders (rare) [13].

Cryptogenic FIRES usually begin with rapid seizures that progress into superrefractory SE. In contrast to infectious encephalitis, its course is usually biphasic with SE following a febrile episode. Moreover, chronic epilepsy directly follows SE without a latent period. The course of cryptogenic FIRES is generally more dramatic with poor seizure control and higher risk of postencephalitic epilepsy [12]. Kothur et al., found that in FIRES-related disorders the levels of cytokines and chemokines in CSF are significantly higher than in encephalitis, suggesting that this elevation is not purely reactive, but rather associated with its aetiology [19]. What differentiates cryptogenic FIRES from autoimmune encephalitis is the lack of other neurological symptoms, such as dyskinesia (typically oro-lingual), behavioural changes (tantrums, irritability), speech and language disintegration and memory deficit [20,21,22,23]. The occurrence of status epilepticus is most characteristic of anti-GABA-B-R (GABA-B receptor) and anti-GABA-A-R (GABA-A receptor) associated encephalitis, however, it may occur in other types of autoimmune encephalitis as well (especially anti-NMDA-receptor associated encephalitis, which is the most common) [24]. Additionally, the course of autoimmune disease is subacute, rather than peracute as in cryptogenic FIRES, and it usually resolves with administration of immunotherapy, however, this may not occur instantly, but rather over a significant period of time. Although cryptogenic FIRES may share similarities with chronic, fever-sensitive epileptic syndromes, such as Dravet syndrome, it usually begins after the fever has ceased. Besides, patients with cryptogenic FIRES typically experience SE only once [2,14], whereas children with Dravet syndrome often experience multiple incidents of SE [25]. FIRES also shows predilection to occur in older children as compared to Dravet syndrome, which first manifestation usually occurs in the infancy. Finally, what differs cryptogenic FIRES from all of these known conditions is lack of specific abnormal findings in multiple diagnostic procedures. Due to this fact, no directed therapy can be implemented and thus treatment is usually unsuccessful.

**Figure 1:**
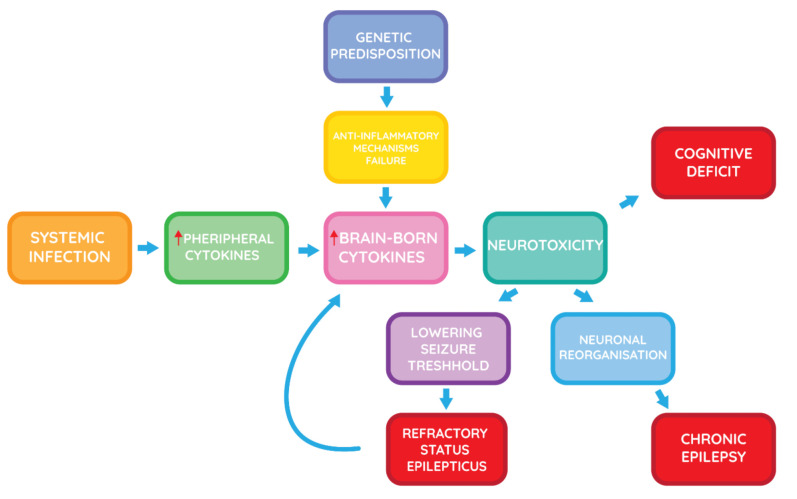
The possible mechanism of immune-mediated reaction i Febrile Infection-Related Epilepsy Syndrome (FIRES) [12,30].

The current hypothesis for causes of cryptogenic NORSE and FIRES is mostly based on immune-mediated mechanisms (Figure 1). Although the clinical presentation matches SE of autoimmune aetiology, the negative results of various antibodies and poor response to immunotherapy contradicts that [12]. However, immunotherapy seems to be more successful in cryptogenic NORSE than in cryptogenic FIRES [26]. It is possible that some cases may be associated with antibodies that have not yet been identified [13]. The clinician could perform exposure of the patient’s CSF to brain slice and then stain for the presence of autoantibody binding to identify the presence of uncharacterised autoimmune encephalitis. The presence of fever prior to the onset of SE suggests, that in FIRES aetiology may be immune-mediated. It has been discussed whether febrile infection, resulting in elevation in levels of pro-convulsant cytokines, may trigger SE in patients with unknown genetic predisposition. Inflammation would act as a factor that lowers seizure threshold, rather than directly cause it [12]. This hypothesis is all the more probable, given that Farias-Moeller et al. had noticed a co-ocurrence of FIRES and hemophagocytic lymphohistiocytosis (HLH), which is a hyperinflammatory disorder [27]. Clarkson et al. conducted a study that suggested that FIRES is associated with reduced expression of endogenous intracellular IL1RA (interleukin-1 receptor antagonist) isoforms and a functional deficiency in IL1RA inhibitory activity [28]. Saitoh et al., suggested that some IL1RA gene mutations may play a role of a predisposing factor, but involvement of multiple genetic factors is probable [29]. This hypothesis may explain possible role of infection likewise. It should be noted that seizure activity may provoke inflammatory activity itself [12].

## 4. Diagnostic Findings in FIRES

### 4.1. Electroencephalogram (EEG)

EEG monitoring should be implemented early and preferably continuously to guide the intensity of implemented therapy and to recognise nonconvulsive status epilepticus [12]. Moreover, it has been noticed that a shifting focal seizure pattern was most predictive of drug-resistant epilepsy occurring in the chronic phase of FIRES [31].

In FIRES, the seizures are usually focal or secondarily generalised without regaining consciousness between them [9,12,14]. In the majority of cases they take the form of eye and head deviation, facial, peribuccal and eyelid myoclonia [2,3,5,9]. Some researchers noticed autonomic symptoms in a few patients [3,16]. Multifocal seizures were observed in most of the patients and in some of them, the foci migrated, indicated that both hemispheres could be affected [2,3]. The first seizure was often prolonged, secondarily generalised convulsive and required barbiturate-induced coma for an average of 3 weeks [5]. Pre-treatment EEG consisted of high-voltage slow background activity, but later patients frequently developed interictal epileptiform discharges with a variety of spatial distribution [3]. The origin of the seizures was most commonly located in temporal, frontotemporal and frontal regions (perisylvian area is often mentioned) [2,9,14]. Overall, FIRES has been characterised by the following EEG findings: (1) beta-delta complexes resembling extreme delta brush (EDB), (2) gradual increase in seizure burden, (3) seizure onset with prolonged focal fast activity, followed by the gradual appearance of well-formed rhythmic spike or spike-and-wave complexes, and (4) shifting ictal activity [32].

### 4.2. MRI

Brain MRI should be performed as soon as possible to exclude any structural causes of status epilepticus. Initial brain MRI is abnormal in 70% of cases in adult NORSE, however, in paediatric patients with FIRES MRI anomalies are usually missing or nonextensive [12]. If these abnormalities do exist, hyperintensities in temporal regions, insula and basal ganglia are noticed the most consistently [2,5,14,33]. A follow-up MRI in chronic phase is rarely normal with generalised brain atrophy reported in almost half of the patients [33]. In some patients the imaging studies presented hyperintensities in temporal regions (including hippocampus) bilaterally [3,9,33]. In some cases the follow-up MRI was normal, even though the initial scan had shown irregularities [12]. 

### 4.3. Cerebrospinal Fluid (CSF)

Cerebrospinal fluid examination does not provide any details suggesting a specific diagnosis. Granulocyte pleocytosis has been observed in over 50% of patients [9]. In some cases, CSF proteins were increased, however, oligoclonal bands are scarcely reported [2,5,9,16]. Some researchers have demonstrated that in FIRES patients the levels of proinflammatory cytokines and chemokines were elevated, proving that intrathecal inflammation is present [19,34]. It is worth mentioning that Sakuma et al. had found that the levels of these mediators were higher in CSF than in serum [34]. No anti-neuronal antibodies have been found in cryptogenic FIRES in children except for a few cases [5,9].

### 4.4. Other Findings

Metabolic evaluations in blood and CSF do not present any abnormalities [5,9]. Immunologic studies for systemic diseases were also negative [9]. The results of genetic testing are mostly negative, with a few heterozygotic sequence variants without pathological evidence in SCN2A and POLG1 genes [11]. Brain biopsies performed in some cases demonstrated gliosis [5,9], but evidence of inflammation was mostly lacking [4,5,9].

## 5. Treatment of FIRES

Treatment with anti-epileptic drugs (ASMs) is usually unsuccessful during the status epilepticus phase of FIRES [5]. In large retrospective study on 77 patients conducted by Kramer et al. patients received a median of six anti-epileptic drugs [9]. Most of the patients required mechanical ventilation with a median length of 41 days [9]. Almost 60% of patients required implementation of burst-suppression coma (BSC) using barbiturates (phenobarbital, pentothal or thiopental) [9]. It should be pointed out that prolonged BSC was associated with longer mechanical ventilation and worse cognitive outcome [35,36], however, it is not clear if the cause of that is medications used for maintaining BSC or rather the extensity of the status epilepticus itself, requiring prolonged coma. Nonetheless, anaesthetic use can temporarily stop seizure activity, but they reoccur as soon as this treatment is ceased [9,12].

Although immunotherapy seems effective in some studies conducted in adult populations [22,26], it has been mostly ineffective in children [2,5,9,16]. This includes steroids (mostly intravenous methylprednisolone), intravenous immunoglobulins (IVIG), plasmapheresis and second-line therapies using anakinra, tocilizumab, tacrolimus, cyclophosphamide and azathioprine [26]. Despite the lack of success, first-line immunotherapy should be considered within the first week nonetheless, provided that immune causes have been recognised the most commonly in NORSE and FIRES [12,13]. The most frequently used doses are 10–30 mg/kg up to 1 g of intravenous methylprednisolone per day for 3–5 days in children [13]. IVIG are typically administered for 5 days at a dose of 0.4 g/kg/day and plasmapheresis is performed 3–5 times on alternate days [13]. With failure of the first-line immunotherapy, the second-line therapy should be implemented. Lai et al. reported that early administration of anakinra was associated with lesser mechanical ventilation days, ICU and hospital length of stay, and possibly seizure reduction [37]. Tocilizumab, a humanised monoclonal antibody against the interleukin-6 receptor (IL-6R), was administered to seven NORSE patients by Jun et al., resulting in termination of SE in six of them (86%) [38]. All of these patients were adult, however Cantarín-Extremera et al. reported two paediatric cases of refractory status epilepticus treated successfully with tocilizumab [39]. Stredny et al., reported a case of successful tocilizumab treatment in a patient with FIRES refractory to anakinra [40].

In ketogenic diet (KD), the majority of the body energy is generated by mitochondrial oxidation of fatty acids. This leads to the synthesis of ketone bodies: acetoacetate and β-hydroxybutrate. These compounds are used by the brain as a source of energy alternative to glucose. Ketogenic diet results in elevation of polyunsaturated fatty acids as well, which alter ion channels regulation leading to hyperpolarisation of neurons [41]. A decrease in glucose consumption and the production of glycolytic ATP may induce ATP-sensitive potassium channels, which also results in hyperpolarisation. This increases the seizure threshold responsible for reduction in epileptic activity [42]. KD is thought to enhance brain energy reserves and energy metabolism genes [41]. Moreover, it results in elevation of inhibition neurotransmitters such as GABA (γ-aminobutyric acid), agmatine, monoamines, galanin and neuropeptide Y [43]. It is noteworthy that KD may have a potential neuroprotective role in seizure-related neuronal damage, which may be a result of long-term epileptic activity in NORSE and FIRES [42,44]. KD implementation in RSE patients is feasible and has a relatively low rate of complications [45]. KD has been described as successful in several paediatric cases of FIRES and is considered as one of the most effective treatments [2,46]. Nabbout et al. reported a success in stopping seizures in seven out of eight patients (88%) who received KD and reached ketonuria. In one patient, whose KD was abruptly cessed, intractable relapse of SE occurred resulting in death of the patient [47].

Cannabidiol (CBD) has been successfully used in treatment of refractory epileptic diseases such as Dravet syndrome and Lennox-Gestaut syndromes when ASMs failed [48]. The mechanism of its anti-epileptic activity is still not fully understood, however, it seems it is multimodal. The possible effects of CBD may include intracellular Ca2+ modulation and adenosine-mediated signalling regulations [49]. Gofshteyn et al., conducted a study on CBD-therapy in FIRES patients with promising results—in six out of seven patients the quantity of seizures reduced, mostly in chronic phase [50]. The dosage was titrated up to 25 mg/kg/d, however, in one patient a significant reduction in seizures was achieved with 15 mg/kg/d [50]. These promising findings may suggest the potential role of CBD in refractory status epilepticus. Nonetheless, further studies are needed.

Therapeutic hypothermia at 33–34 °C in NORSE and FIRES can possibly play a role in neuroprotection and anti-edematous effect in damage caused by extensive epileptic activity. It has been shown to improve outcome in children with FIRES [51,52]. However, further studies are needed to confirm these findings.

Whether vagal nerve stimulation (VNS) is successful in FIRES and NORSE still requires further studies, however, in recent studies VNS has shown to have a favourable impact on SE in children [53].

**Figure 2:**
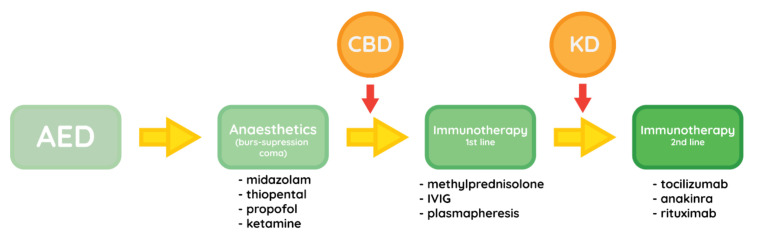
Most commonly used therapeutic strategy for managing the acute phase of FIRES.

All the above-mentioned procedures and medications regard the acute phase of FIRES. The most common strategy is shown in Figure 2. Managing extensive epileptic activity is crucial for the long-term health and cognitive functions in the rest of the patient’s life. In the chronic phase the treatment is mostly symptomatic, as the damage done in the status epilepticus does not progress further. Despite that, epilepsy that is usually associated with the chronic phase is very difficult to treat, with frequent need for polytherapy. The first-line therapy seems to consist of KD, clobazam, phenobarbital and potentially cannabidiol [12].

Treatment in FIRES patients is difficult and often frustrating. Desperate parents of the affected child insist on trying out different therapeutic approaches, which have not yet been proven to be successful. This process may feel like grasping at straws, as the patients’ well-being is an utmost priority for the clinician. Still to this day, we lack a specific and effective treatment that would prevent the disastrous neuronal damage.

## 6. Outcome of FIRES

The overall outcome of FIRES is unfavourable—the mortality rate ranges from 9% to 25% [5,15]. Of those that survive, 93% were left with refractory epilepsy that occurred without any latent period or in a maximum of 3 months following the status epilepticus [9]. The seizures were mostly focal or secondarily generalised [9]. Only 24% of patients had normal or borderline cognitive level [9]. It is worth mentioning that the level of cognitive dysfunction was associated with longer duration of burst-suppression coma and younger age at onset of FIRES [9].

## 7. Case Report

A previously healthy, well-developing 4-year-old boy presented fever (up to 39.5 °C) and signs of upper respiratory tract infection, which was treated symptomatically at first. With no improvement, the general practitioner (GP) prescribed him oral phenoxymethylpenicillin for his illness. After 4 days of antibiotic treatment, the parents decided to report to the hospital as the boy’s condition was deteriorating and petechial rash. His saturation dropped to 44% at times; bradycardia and consciousness disorder occurred. The child was immediately admitted to ICU, where mechanical ventilation, thiopental sedation and broad-spectrum intravenous antibiotic (ceftriaxone and amikacin) treatment (as well as acyclovir) were implemented. On the same day, lip twitching, downward eyeball rotation and loss of own respiratory drive were observed. Status epilepticus had been observed since the 3rd day of hospitalisation (despite thiopental sedation), which manifested firstly with generalised tonic-clonic convulsions. Electroencephalogram showed almost constant epileptiform multifocal discharges: spikes, polyspikes, sharp waves, spike–wave complexes, sharp and slow wave complexes. Apart from seizures with electrophysiological correlation, there were also stimulus-induced rhythmic, periodic, or ictal discharges (SIRPID).

During this time the patient was still feverish (with his temperature rising up to 38 °C). In the following days, the seizures were still present, in spite of extensive modification of the antiepileptic therapy. Above many ASMs, the patient received clonazepam (max 2 mg/kg), phenytoin (i.v; 10 mg/kg), levetiracetam (i.v; up to 80 mg/kg), valproate (i.v.; 40 mg/kg), clobazam (i.v.;1.3 mg/kg) and topiramate (1 mg/kg administered through percutaneous endoscopic gastrostomy—PEG) and diazepam (0.6 mg/kg). Seizures occurred independently or were provoked by touch stimuli (e.g., during nursing procedures). Constant infusion of phenobarbital was administered. To stop convulsion, diazepam, midazolam, propofol and cisatracurium were used. Seizure frequency increased and thus the decision of immunotherapy implementation was made. The patient was administered glucocorticoids on the 9th day of the patient’s hospital stay. Due to no therapeutic success of this therapy, the IVIG infusion was implemented. The patient received 2 g/kg of IVIG in the course of 4 days, starting on the 13th day of his hospital stay. On a later date plasmapheresis was performed on five consecutive days, and IVIG therapy was repeated. However, with no noticeable improvement. The patient also received anakinra on the 42nd day of hospitalisation, without any success. With epileptic activity constantly present, CBD oil was administered, without a drastic change. It seemed that the best response was achieved with intravenous thiopental and clonazepam. Weaning off thiopental at first resulted in reoccurrence of epileptic activity. Seizure frequency gradually ceased, regardless of the implemented therapy. We consider discontinuation of thiopental as the definite end of status epilepticus. This overall took 12 weeks.

During this acute phase the semiology of epileptic seizures was changing. The patient presented generalised tonic-clonic convulsions, myoclonia, tonic seizures, rhythmic limb movement, eyeball rotation. With clear epileptic seizures cessation, more extrapyramidal dystonia appeared, especially provoked by touch stimulus. During this episode a rise in body temperature was observed. The patient received gabapentin (40 mg/kg administered through PEG) as these episodes were thought to be caused by pain without desired improvement. Due to hypertonia and excessive dystonia, baclofen therapy was implemented with an intrathecal pump (in the dose of 99 μg per day).

At first, the neurological examination was normal, except epileptic activity. With time, hypertonia in limbs, ankle clonus and Babinski sign appeared. Axial hypotonia was present. At times nystagmus and oral automatism were observed. The patient never regained full consciousness. His eyes remained open, but little to no reaction to external stimuli was detected.

Diagnostic procedures did not carry any suggestions on what could be the cause of this dreadful condition. Initial CT scan was normal, though MRI performed at a later date suggested encephalitis. CSF study revealed pleocytosis with high level of granulocytes and elevation in protein concentration, but oligoclonal bands were absent. However, no etiological factor of possible infection was identified. All immunologic testing was negative, including antineuronal antibody findings (Western blot performed from a plasma sample). Metabolic studies were all negative. Further MRI imaging revealed progressive neurodegeneration and brain atrophy, especially in hippocampal regions. The cerebral ventricle appeared to be enlarged and surrounded by areas of microvascular leakage and reduced venous blood flow. The patient’s cerebral cortex appeared to be atrophic. A thrombus located in the left sigmoid sinus was observed, however, blood flow was preserved. The diagnosis of FIRES was made after extensive differential diagnosis was performed (Table 1).

During his 9-months hospitalisation, the patient developed cholelithiasis, and thus surgery was required. Due to this fact ketogenic diet could not be implemented in this patient. In the course of hospital stay, the patient contracted SARS-CoV2 infection which resulted in the need for oxygen therapy. The patient still experienced multiple epileptic seizure which he received clonazepam for. Long-term cognitive level was poor, tending towards vegetative state.

## 8. Conclusions

NORSE and FIRES are catastrophic conditions with many complications. In paediatric surroundings, the dreadful presentation of refractory status epilepticus in previously healthy children is especially traumatising for the parents. With recent hopeful scientific reports, treating FIRES and preventing its adverse effects on developing brains may become possible in many cases in the future. We described a case with an unusual presentation of the chronic phase, in which dystonia and extrapyramidal symptoms seem to be the biggest problem.

## Figures and Tables

**Table 1:** Differential diagnosis of the patient presented in this paper [12].

Diagnosis	Pros	Cons
Cryptogenic FIRES	Previously healthy and well-developing child Uninformative family history No infectious agent identified RSE following febrile infection No latent period between RSE and chronic epilepsy Brain atrophy in MRI imaging with predilection towards hippocampal regions Resistance to ASMs and anaesthetics	Severe course of febrile illness prior to the occurrence of SE Initial MRI findings suggesting encephalitis Elevated protein concentration in CSF Domination of dystonia in chronic clinical presentation
Infectious encephalitis	RSE following febrile episode MRI suggesting encephalitis	No improvement after implementation of antibiotic and acyclovir treatment No identified infectious agent Biphasic course Epilepsy without a latent period after SE
Metabolic disease	Long duration of excitation Basal ganglia involvement (dystonia) Brain atrophy in imaging studies	Late age of onset (4 years) No laboratory findings suggesting metabolic disorder
Autoimmune encephalitis	Interval between occurrence of febrile episode and RSE Elevated protein concentration and pleocytosis in CSF	Peracute course of disease No memory deficit, behavioural changes or psychosis observed earlier No oligoclonal bands in CSF Negative antibody findings No response to immunotherapy

## Data Availability

The datasets generated during and/or analyzed during the current study are available from the corresponding author on reasonable request.
